# Under-diagnosis of pain by primary physicians and late referral to a palliative care team

**DOI:** 10.1186/1472-684X-11-7

**Published:** 2012-06-07

**Authors:** Masako Akashi, Eiji Yano, Etsuko Aruga

**Affiliations:** 1 Department of Palliative Medicine, Teikyo University Hospital, 2-11-1 Kaga Itabashi ku, Tokyo, 173-8605, Japan; 2 Department of Hygiene and Public Health, Teikyo University School of Medicine, 2-11-1 Kaga Itabashi ku, Tokyo, 173-8605, Japan

**Keywords:** Palliative care team, Pain, Primary physician, Cancer, Referral, Concordance

## Abstract

**Background:**

Under-diagnosis of pain is a serious problem in cancer care. Accurate pain assessment by physicians may form the basis of effective care. The aim of this study is to examine the association between late referral to a Palliative Care Team (PCT) after admission and the under-diagnosis of pain by primary physicians.

**Methods:**

This retrospective study was performed in the Teikyo University teaching-hospital for a period of 20 months. We investigated triads composed of 213 adult cancer inpatients who had coexisting moderate or severe pain at the initial PCT consultation, 77 primary physicians, and 4 palliative care physicians. The outcome of the present study was the under-diagnosis of pain by primary physicians with routinely self-completed standard format checklists. The checklists included coexisting pain documented independently by primary and palliative care physicians at the time of the initial PCT consultation. Under-diagnosis of pain was defined as existing pain diagnosed by the palliative care physicians only. Late referral to PCTs after admission was defined as a referral to the PCT at ≥20 days after admission. Because the two groups displayed significantly different regarding the distributions of the duration from admission to referral to PCTs, we used 20 days as the cut-off point for “late referral.”

**Results:**

Accurate pain assessment was observed in 192 triads, whereas 21 triads displayed under-diagnosis of pain by primary physicians. Under-diagnosis of pain by primary physicians was associated with a longer duration between admission and initial PCT consultation, compared with accurate pain assessment (25 days versus 4 days, *p* < 0.0001). After adjusting for potential confounding factors, under-diagnosis of pain by the primary physicians was significantly associated with late (20 or more days) referral to a PCT (adjusted odds ratio, 2.91; 95% confidence interval, 1.27 − 6.71). Other factors significantly associated with under-diagnosis of pain were coexisting delirium and case management by physicians with < 6 years of clinical experience.

**Conclusions:**

Under-diagnosis of pain by primary physicians was associated with late referral to PCTs. Shortening the duration from admission to referral to PCTs, and increasing physicians’ awareness of palliative care may improve pain management for cancer patients.

## Background

It is commonly believed that 75% of patients with cancer will have pain at some point in their disease process and that adequate pain management can be achieved through simple measures in 85 − 95% of cases [1,2]. However, at least 40% of cancer patients are reported to receive inadequate analgesia [3,4]. Palliative Care Teams (PCTs) provide care, including pain management in acute-care hospitals during the early course of the disease, in conjunction with other life-prolonging therapies, such as chemotherapy or radiation therapy. PCTs facilitate collaboration among specialists and the early introduction of palliative care services.

It has been reported that accurate pain assessment by physicians is associated with improved outcomes for pain management [5-8]. In addition; early referral to palliative care is an important indicator of the quality of care for pain management [9]. Therefore, we hypothesized that early referral to a PCT would be associated with accurate pain assessment by primary physicians.

In previous studies, the barriers to pain assessment have been examined from a variety of perspectives, including barriers related to patients and health care professionals [10]. The most significant barrier was a patient’s inability to report pain owing to dementia, delirium, and depression [11]. Physician-related barriers may result from insufficient knowledge of palliative care [12]. However, these studies were conducted between primary physicians and oncologists, excluding palliative care physicians [13,14]. Although palliative care physicians have more opportunity to assess cancer patient pain in an inpatient setting, to our knowledge, few studies have compared the specific barriers to accurate pain assessment between primary and palliative care physicians. Moreover, the relationship between late referral to a PCT and the under-diagnosis of pain by primary physicians has not been assessed.

The aim of the present study was to assess the relationship between late referral to a PCT after hospital admission and the under-diagnosis of pain by primary physicians in Japan, which may help to identify the optimal time to consult with a PCT for pain assessment.

## Methods

### Study design, setting, and samples

We retrospectively examined the relationship between the duration from admission to initial PCT consultation and under-diagnosis of pain by primary physicians. We reviewed the electronic medical records of 351 consecutive cancer inpatients who had been referred to the PCT between June 2009 and March 2011. Our study samples comprised triads of patients and their primary and palliative care physicians at the initial PCT consultation.

The present study was conducted according to the principles of the Declaration of Helsinki. The study protocol was reviewed and approved by the Institutional Review Board and the Ethics Committees of Teikyo University.

#### Setting

We conducted this study at Teikyo University Hospital, in Japan, which is a teaching-hospital with 24 medical departments and 1154 beds, providing general acute care. The Department of Palliative Care at the hospital has provided PCT services since April 2009.

#### Patients

We retrieved data from all consecut ive cancer inpatients over 18 years of age and with moderate to severe pain who were referred to the PCT of the hospital by their primary physicians during a 20-month period. Patients who were referred to the PCT on two or more occasions, and those without moderate or severe pain were beyond the scope of this study and were excluded from the study. We defined coexisting moderate or severe pain as that rated by patients at an intensity of pain was either ≥ 4 on the Numerical Rating Scale (NRS), or ≥ 8 on the Abbey Pain Scale (APS), documented by palliative care physicians [15,16].

#### Physicians

All primary physicians (full-time employed, including residents) who referred a selected patient to the PCT were included in the study.

The PCT comprised three palliative care physicians, one psycho-oncology physician, and two nurse practitioners. The service provided by the PCT was primarily consultative and was available to all inpatients upon request by a patient’s primary physician. The PCT conducted daily rounds and participated in decision-making for the treatment program, critical care, nursing, respiratory therapy, and nutritional service. At the initial PCT consultation, the palliative care physicians assessed the referred patients, proposed problems, and organized possible solutions.

### Outcome: under-diagnosis of pain by primary physicians

Primary and palliative care physicians independently recorded each patient’s problems using the same standardized checklist (i.e., coexisting pain: Yes or No) at the initial PCT consultation. As cancer pain is generally chronic, and given that the mean interval between referral to the PCT and the initial medical interview by the PCT was 0.7 days, we considered the assessment of cancer pain by primary and palliative care physicians to have been performed at the same time. In addition, primary physicians documented the reason for referral to a PCT, and the intensity and locations of pain were documented by primary physician. The form for palliative care physicians comprised three parts; patients’ checklists documented by palliative care physicians, characteristics of pain rated by patients, and assessment and therapy plan documented by palliative care physicians. The characteristics of pain, such as a diagram of locations of pain, and intensity of pain as measured in the patient’s marks on the NRS, were based on the Brief Pain Inventory (BPI) [17]. For patients who could not verbalize, palliative care physicians assessed the patients’ pain using the APS instead of the NRS [16] as rated by patients. The palliative care physicians considered the characteristics of pain in their assessments and therapy plans. The data recorded included the reason for consultation, the demographics of the patients, and the history of illness.

To directly compare the assessments of primary and palliative care physicians, we defined accurate pain assessment as the identification of existing pain by both primary and palliative care physicians using the standard format at the time of the initial PCT consultation. Under-diagnosis of pain was defined as the identification of pain by only palliative care physicians.

#### Exposure: interval between admission and the initial PCT consultation

Various definitions of “palliative care consultation” or “referral” have been proposed [17,18]. The present study defined referral to the PCT as receipt by the PCT of documents requesting advice or assistance in directing patient management that were signed by the physician who was primarily responsible for the care of the patient. We defined an interval of 20 days between hospital admission and initial PCT consultation as the cut-off point between early and late referral. Because time between early and late referral was significantly different and had a non-normal distributions, we performed a dichotomous rather than continuous analysis.

### Covariates

Covariates that can affect pain assessment by a physician include patient demographics, such as age (continuous), gender, primary cancer site, Karnofsy Performance Status (KPS), therapy status, purpose of admission, current opioid use at the initial PCT consultation, duration of hospitalization, coexistence of delirium, as well as physician characteristics, such as years of experience (<6, 6–10, >10 years), and clinical department. Current opioid use at the initial PCT consultation has been shown to affect the prescription of opioids by a primary physician and to reflect a physician’s knowledge of palliative care [12].

From 2003 to 2006, the Cancer Control Act was established to improve the quality of life for all cancer patients in Japan, and disseminating the knowledge of palliative care among physicians was identified as an important area of improvement. Since the Act took effect, palliative care has been a part of medical education, and so physicians with 6–10 years of experience have studied palliative care as medical students. Therefore, we used this group of physicians as a reference. The coexistence of delirium was diagnosed by a psycho-oncology specialist, who was a member of the PCT, using the Diagnostic and Statistical Manual of Mental Disorders (DSM-IV) criteria. Clinical departments were divided into three categories based on clinical experience related to cancer patients, as collected from the database of cancer patients registered at the hospital in 2009. As the physicians’ gender was not reported with regard to barriers to pain assessment, it was excluded from the covariates.

### Statistical analysis

First, we summarized the baseline demographics of the patients and physicians, and the symptom profiles, including percentages and medians for clinical variables. Second, the results of the baseline assessment were compared according to the two categories of pain assessment: accurate pain assessment and under-diagnosis of pain by primary physicians. Comparisons were made using the Wilcoxon rank-sum test for continuous variables and the chi-square test or Fisher’s exact test for categorical variables, depending on the variable type and data distribution.

Third, logistic regression models were used to assess the relationship between late referral to the PCT and the risk for under-diagnosis of pain after adjusting for covariates. The results were shown as the odds ratio (OR) and 95% confidence interval (CI). No multicollinearity was observed among the independent variables. Values of *P* < 0.05 (two-sided) were considered to indicate statistical significance. All analyses were performed using SAS software (Windows Version, Release 9.02; SAS Institute, Cary, NC, USA).

## Results

### Baseline characteristics

#### Patients

**Figure 1:**
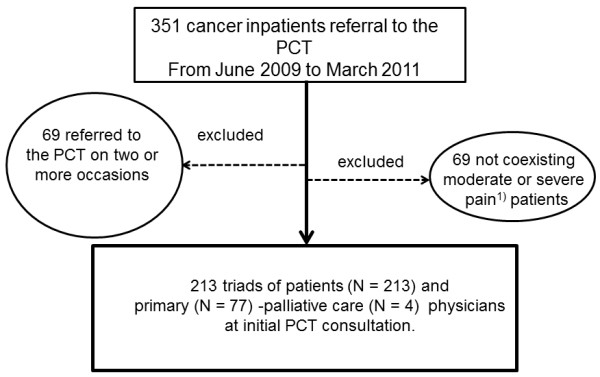
**Patients in this study.** PCT; Palliative Care Team 1) We defined moderate or severe pain as intensity of pain was rated ≧ 4 on the Numerical Rating Scale (NRS) by patients, or documented ≧8 on the Abbey Pain Scale (APS) by palliative care physicians at the initial consultation to a PCT.

Of the 351 hospitalized patients consecutively referred to a PCT during the study period, 69 were excluded because they had been referred to the PCT on two or more occasions, and another 69 patients were excluded because they did not have moderate or severe pain (Figure 1). The remaining 213 patients and their primary and palliative care physicians were included in the final analysis. No data were missing for the 213 patients assessed. The demographics of the patients are presented in Table 1. The median interval between admission and initial PCT consultation was 5 days (range, 0–251).

**Table 1:** **Characteristics of the patients at the initial PCT consultation (*n*=213)**

**Characteristics**	**Number**	**Percentage**
Age		
Median (Range)	68 (22-94)	
Gender		
Male	123	58
Female	90	42
KPS		
Median (Range)	40 (10-80)	
Primary cancer site		
Respiratory tract	32	15
Gastrointestinal tract and liver/galbladder/pancreas	59	28
Genitourinary	77	36
Others	45	21
Treatment status at the initial PCT consultation		
Chemotherapy/Radiotherapy/Surgery/Diagnosis	103	49
Only symptom management	110	51
Purpose of admission		
Chemotherapy/Radiotherapy/Surgery/Diagnosis	86	40
Only symptom management	127	60
Coexistence of delirium		
Yes	25	12
No	188	88
Opioids use at the initial PCT consultation		
Yes	93	43
No	120	57
Duration of hospitalization (Days)		
Median (Range)	34 (2-394)	
Interval between admission and initial PCT consultation (Days)		
Median (Range)	5 (0-251)	

*PCT*; Palliative Care Team.

*KPS*; Karnofsy Performance Scale.

#### Physicians

Table 2 shows the characteristics of the physicians in the study (primary, *n* = 77; palliative care, *n* = 4). The majority of the primary physicians (81%) were male and 40% had been practicing medicine for 6–10 years. The primary physicians had consulted with the PCT 3.7 ± 0.6 times (mean and standard deviation).

**Table 2:** Characteristics of primary and palliative care physicians

**Characteristics**	**Primary physicians (N = 77)**		**Palliative care physicians (N = 4)**	
	**Number**	**Percentage**	**Number**	**Percentage**
Gender				
Male	62	81	1	25
Female	15	19	3	75
Clinical department				
Internal medicine less-experienced oncology ^1)^	11	14	0	0
Internal medicine more-experienced oncology ^2)^	23	30	0	0
Surgery ^3)^ and Urology/Obstetrics and Gynecology	31	40	0	0
Others ^4)^	12	16	4	100
Experience as physicians				
< 6years	21	27	1	25
6−10years	31	40	1	25
> 10years	25	33	2	50

^1)^ General medicine, Internal medicine specialized Renal and Cardiovascular.

^2)^ Internal medicine specialized Gastroenterological, Respiratory, Hematology, and Oncology.

^3)^ Surgery specialized Upper and Lower gastroenterological, Hepato-Biliary-Pancreatic Surgery, Respiratory, Mammary gland, and Thyroid.

^4)^ Orthopedic surgery, Otorhinolaryngology, Dermatology, and Oral surgery.

Less-experienced and more-experienced oncology was defined by the data from the cancer patient hospital register.

### Under-diagnosis of pain by primary physicians

The majority of patients (91%) were referred to the PCT for advice regarding symptom management. The rate of diagnosis of pain by both primary and palliative care physicians was 66%. These findings were nearly the same as those of previous studies [19].

The relationships between triads characteristics and pain assessment by primary physicians are shown in Table 3. Accurate pain assessment was significantly associated with early referral to the PCT compared with under-diagnosis of pain (4 days versus 25 days, *p* < 0.0001). Physicians with clinical cancer experience used the NRS to assess the pain intensity. Neither clinical departments (Tables 3 and 4) nor current use of analgesia or opioids was associated with the under-diagnosis of pain by primary physicians.

**Table 3:** Characteristics of triads of patient-physician, by two categories of accurate pain assessment and under-diagnosis of pain by primary physicians

	***Coexisisting moderate or Severe pain6) (N = 213)***				
	***Accurate pain assessment (N = 192)***		***Under-diagnosis of pain by primary physicians (N = 21)***		
***Variables***	***Number***	***Percentage***	***Number***	***Percentage***	***p-value*****†**
Age					
Median (Range)	68 (22-94)		65 (41-82)		0.71
Gender					
Male	112	52.6	11	5.2	0.60
Female	80	37.5	10	4.7	
KPS					
Median (Range)	40 (10-80)		40 (10-80)		0.79
Primary cancer site					
Respiratory tract	29	13.5	3	1.4	0.98
Gastrointestinal tract and liver/galbladder/pancreas	53	24.9	6	2.8	
Genitourinary	70	32.9	7	3.3	
Others	40	18.8	5	2.4	
Treatment status at initial PCT consultation					
Chemotherapy/Radiotherapy/Surgery/Diagnosis	95	44.6	8	3.8	0.32
Only symptom management	97	45.5	13	6.1	
Purpose of admission					
Chemotherapy/Radiotherapy/Surgery/Diagnosis	77	35.7	10	4.7	0.48
Only symptom management	115	54.4	11	5.2	
Coexistence of delirium					
Yes	21	9.9	4	1.9	0.27
No	171	80.2	17	8.0	
Current opioid use at initial PCT consultation					
Yes	83	39.0	9	4.2	0.97
No	109	51.1	12	5.7	
Duration of hospitalization (Days)					
Median (Range)	34 (2-394)		42 (8-293)		0.06
Interval between admission and initial PCT consultation (Days)					
Median (Range)	4 (0-148)		25 (0-251)		< 0.0001**
Clinical department of primary physician					
Internal medicine less-experienced oncology ^1),5)^	41	19.3	7	3.3	0.33
Internal medicine more-experienced oncology ^2),5)^	66	31.0	7	3.3	
Surgery^3)^ and Urology/Obstetrics and Gynecology	65	30.5	7	3.3	
Others^4)^	20	9.4	0	0	
Experience of primary physician					
< 6years	22	10.4	3	1.4	0.17
6-10years	81	38.0	13	6.2	
> 10years	89	41.8	5	2.3	

* *p*<0.05.

**p<0.01.

*** *p*<0.001.

†Compared according to the two categories of pain assessment: accurate pain assessment and under-diagnosis of pain by primary physicians.

†Wilcoxon rank-sum test for age, KPS, duration of hospitalization, and interval between admission and initial consultation to PCT;χ² for gender, primarycancer site, tratment status at initial PCT consultation, purpose of admission, coexistence of delirium, current opioid use at initial PCT consultation, durationof hospitalization, interval between admission and initial PCT consultation, clinical departments of primary physician, and experience of primary physician.

^1)^ General medicine, Internal medicine specialized Renal and Cardiovascular.

^2)^ Internal medicine specialized Gastroenterological, Respiratory, Hematology, and Oncology.

^3)^ Surgery specialized Upper and Lower gastroenterological, Hepato-Biliary-Pancreatic Surgery, Respiratory, Mammary gland, and Thyroid.

^4)^ Orthopedic surgery, Otorhinolaryngology, Dermatology, and Oral surgery.

^5)^ Less-experienced and more-experienced oncology was defined by cancer patient data from the hospital register.

^6)^ We defined coexisting moderate or severe pain as intensity of pain was ≧ 4 on the Numerical Rating Scale (NRS) rated by patients, or ≧8 on the AbbeyPain Scale (APS) documented by palliative care physicians with the form for palliative care physicians at the initial consultation to a PCT.

*PCT*; Palliative Care Team.

*KPS*; Karnofsy Performance Stasus.

**Table 4:** Multivariate odds ratios for the association of under-diagnosis of pain by primary physicians and independent variables

***Variables***	***Crude OR (95% CI)***	***Adjusted OR*****†*****(95% CI)***
Age	0.99 (0.96-1.03)	0.98 (0.96-1.01)
Gender		
Male	0.79 (0.31-1.94)	0.94 (0.49-1.83)
Female	1.00 (Reference)	1.00 (Reference)
KPS < 40	1.35 (0.53-3.39)	1.10 (0.51-2.34)
≧ 40	1.00 (Reference)	1.00 (Reference)
Primary cancer site		
Respiratory tract	0.83 (0.18-3.74)	0.58 (0.19-1.66)
Gastrointestinal tract and Liver/Gallbladder/Pancreas	0.91 (0.26-3.18)	0.70 (0.25-2.01)
Genitourinary	0.80 (0.24-2.69)	0.43 (0.13-1.40)
Others	1.00 (Reference)	1.00 (Reference)
Treatment status at initial PCT consultation		
Chemotherapy/Radiotherapy/Surgery/Diagnosis	0.63 (0.25-1.58)	1.47 (0.69-3.14)
Only symptom management	1.00 (Reference)	1.00 (Reference)
Purpose of admission		
Chemotherapy/Radiotherapy/Surgery/Diagnosis	1.39 (0.56-3.43)	1.17 (0.56-2.45)
Only symptom management	1.00 (Reference)	1.00 (Reference)
Coexistence of delirium		
Yes	1.92 (0.59-6.23)	2.92 (1.23-6.94)**
No	1.00 (Reference)	1.00 (Reference)
Current opioid use at initial PCT consultation		
Yes	0.98 (0.39-2.45)	0.84 (0.43-1.63)
No	1.00 (Reference)	1.00 (Reference)
Duration of hospitalization (Number of days)		
	1.01 (0.99-1.02)	0.99 (0.98-1.01)
Interval between admission and initial PCT consultation (Days)		
> 20 days	3.06 (1.65-5.69)**	2.91 (1.27-6.71)**
≦ 20 days	1.00 (Reference)	1.00 (Reference)
Clinical department of primary physician ^1)^		
Internal medicine less-experienced oncology ^2), 5)^	1.21 (0.13-11.06)	1.51 (0.38-5.97)
Internal medicine more-experienced oncology ^3), 5)^	1.33 (0.16-11.37)	1.81 (0.42-7.76)
Surgery ^4)^ and Urology/Obstetrics and Gynecology	1.00 (Reference)	1.00 (Reference)
Experience of primary physician		
< 6 years	3.45 (1.42-8.36)*	3.51 (1.32-9.35)*
6−10 years	1.00 (Reference)	1.00 (Reference)
> 10 years	1.93 (1.01-3.69)*	1.96 (0.94-4.08)

Under-diagnosis of pain by primary physicians = 1, accurate pain assessment = 0.

† Adjusted for age, gender, KPS, primary cancer site, treatment status at initial PCT consultation, purpose of admission, coexistince of delirium, current opioid use at initial PCTconsultation, duration of hospitalization, interval between admission and initial PCT consultation, clinical department, experience of primary physician.

^1)^ Others was deleted as it was the minority. Others were Orthopedic surgery, Otorhinolaryngology, Dermatology, and Oral surgery.

^2)^ General medicine, Internal medicine specialized Renal and Cardiovascular.

^3)^ Internal medicine specialized Gastroenterological, Respiratory, Hematology, and Oncology.

^4)^ Surgery specialized Upper and Lower gastroenterological, Hepato-Biliary-Pancreatic Surgery, Respiratory, Mammary gland, and Thyroid.

^5)^ Less-experienced and more-experienced oncology was defined by cancer patient data from the hospital register.

*OR*; Odds Ratio.

*CI*; Confidence Interval.

*KPS*; Karnofsy Performance Status.

*PCT*; Palliative Care Team.

*p < 0.05.

**p < 0.01.

We performed a multiple logistic regression analysis for the effect of late referral to the PCT on under-diagnosis of pain. After adjusting for patient age, gender, KPS, primary cancer site, treatment status, purpose of admission, coexistence of delirium, duration of hospitalization, current opioid use at the initial PCT consultation, primary physician clinical department, and primary physician experience, the analysis revealed that late referral to the PCT was significantly associated with an under-diagnosis of pain (OR, 2.91; 95% CI, 1.27 − 6.71; Table 4). Furthermore, years of experience of primary physician (<6 years: OR 3.51, 95% CI 1.32 − 9.35) and coexistence of delirium (OR 2.92, 95% CI 1.23 − 6.94) were significant predictors for under-diagnosis of pain by primary physicians.

## Discussion

The main finding of the prese nt study was that under-diagnosis of pain by primary physicians was associated with a long duration between admission and the initial PCT consultation. Patients who were referred to the PCT more than 20 days after admission were 2.91 times more likely to have experienced under-diagnosed pain by primary physicians than those referred earlier. This association was independent of age, gender, KPS, primary cancer site, treatment status, purpose of admission, coexistence of delirium, current opioid use, duration of hospitalization, clinical department, and years of experience of the primary physician. To our knowledge, few studies have demonstrated a relationship between late referral to the PCT and under-diagnosis of pain. These results support previous studies that showed early referral to palliative care was related to improved quality of care [9,18].

The World Health Organization defines palliative care as “an approach that improves the quality of life of patients and their families facing problems associated with life-threatening illnesses,” and states that this is achieved “through the prevention and relief of suffering by means of early identification and impeccable assessment and treatment” [20]. Although palliative care is rooted in the compassionate care of dying patients, its primary aim is to minimize patient and family suffering at all stages of a life-threatening illness [20]. In a recent randomized control study examining the effects of referral to a PCT during early stages of cancer, Temel et al. found that referral to a PCT in the early stages of the disease led to significantly improved quality of life as well as increased survival [21].

In the present study, late referral to a PCT was associated with the under-diagnosis of pain by primary physicians, and thus a long duration of hospitalization preceded the late referral to a PCT. As patients with advanced cancer deteriorate over time, they may develop a state of unconsciousness, such as delirium. Delirium and psychological problems may contribute to the under-diagnosis of pain. We found that delirium was more common in patients with later referrals to the PCT than in those who received early referrals, and that patients with no delirium were significantly associated with the under-diagnosis of pain (OR 2.92, 95% CI 1.23 − 6.94; Table 4). Although pain assessment scales and reporting techniques for patients who are unable to self-report or who possess cognitive impairment have improved, these scales are not routinely used by primary physicians [22]. A long duration between hospital admission and discharge was not associated with the under-diagnosis of pain by primary physicians, suggesting that a long duration between admission and referral to the PCT, and not a long duration of hospitalization, was critically affected by the accuracy of pain assessment by primary physicians.

However, we considered the possibility that the under-diagnosis of pain would lead to late referral to the PCT. Previous studies have investigated patient’s and physician’s factors related to the under-diagnosis of pain [10-12]. The patients’ factor was the inability to report pain owing to unconsciousness, and the physician’s factor was insufficient knowledge of palliative care [6,23]. The results of the present study, we agree with these findings, showing that patients’ delirium and primary physicians’ inexperience were associated with the under-diagnosis of pain. Physicians with less than 6 years of experience had a risk for under-diagnosing pain that was 3.5 times the risk of physicians who had 6–10 years of experience (Table 4). However, current opioid use, which is related to a physicians’ knowledge of palliative care, was not associated with the under-diagnosis of pain in the present study (Tables 3 and 4), suggesting that other factors contributed to the under-diagnosis of pain. Our finding that late referral to the PCT was associated with the under-diagnosis of pain by primary physicians has not been previously reported and makes a unique contribution to the literature.

Previous studies have reported that early referral to PCTs is beneficial to cancer patients, however, physicians usually refer patients to specialized palliative care programs in the very late stages of cancer [9,18]. Although physicians state that patients should ideally receive hospice care for 3 months prior to death [24], the majority of patients survive less than 1 month under hospice care [25,26]. The most effective method to shorten the duration between admission and the initial PCT consultation has not been determined. Thus, we recommend that methods designed to shorten this duration to assess pain accurately, regardless of level of knowledge of palliative care, be further explored.

### Limitations of the study

The present study has several limitations. First, this study was conducted at a single institution using a retrospective design. Nevertheless, we believe our findings can be generalized to numerous hospitals and physicians. Although our study included a homogenous study population, a low exclusion rate, and an adjustment for important confounders, the nature and number of problems documented at the initial PCT consultation did not differ from those reported in previous studies [27]. Furthermore, our results cannot be generalized beyond the study subjects who were referred to a PCT. There are two possible explanations for primary physicians not to refer their patients to a PCT. First, the primary physicians may not recognize the pain. If we were to include this type of patient in our study, the association between under-diagnosis and late referral to a PCT would be stronger. Second, the primary physician may be able to appropriately manage the pain and thus would not need to refer the patient to a PCT. For this case, there would be no relationship between under-diagnosis and late referral to a PCT. As previous studies have reported that early referral to hospice care improved symptom management, we believe that early referral to palliative care would have benefited patients who were not referred to PCTs.

Moreover, we did not directly measure the physicians’ knowledge of palliative care which is considered a factor in the under-diagnosis of pain. However, physicians who had been practicing for 6–10 years, and thus had been trained in palliative care after 2003, tended to refer patients to the PCT sooner and generally displayed greater knowledge of palliative care. Thus, a physician’s years of experience served as a surrogate for knowledge of palliative care in the present study.

Finally, we did not consider the strength and type of pain experienced by patients. The effects of these variables on accurate pain assessment should be prospectively evaluated in a future study.

## Conclusions

Despite these limitations, this study presents findings with important implications and suggests the benefits of early referral to a PCT, as a long duration between admission and initial PCT consultation was associated with under-diagnosis of pain of cancer inpatients. These findings emphasize the need for earlier referral to PCTs for accurate pain assessment for primary physicians.

## Competing interests

The authors declare that they have no competing interests.

## Authors’ contributions

MA, EY, and EA designed the study and drafted the paper. MA led the data collection and analysis. MA wrote the paper. All authors contributed to the paper, reviewed drafts and approved the final content. All authors read and approved the final manuscript.

## Pre-publication history

The pre-publication history for this paper can be accessed here:

http://www.biomedcentral.com/1472-684X/11/7/prepub
